# Boosting the efficiency of single junction kesterite solar cell using Ag mixed Cu_2_ZnSnS_4_ active layer†

**DOI:** 10.1039/c7ra12352c

**Published:** 2018-01-29

**Authors:** Uday Saha, Md. Kawsar Alam

**Affiliations:** Department of Electrical and Electronic Engineering, Bangladesh University of Engineering and Technology Dhaka 1205 Bangladesh kawsaralam@eee.buet.ac.bd kawsar.alam@alumni.ubc.ca

## Abstract

We propose a silver (Ag) mixed Cu_2_ZnSnS_4_ (ACZTS) based solar cell architecture to improve the efficiency of single junction Cu_2_ZnSnS_4_ (CZTS) solar cells. The configuration exploits enhancement of depletion region using a CdS/ACZTS/CZTS architecture. The doping concentration of different layers is adapted such that the primary absorber layer (ACZTS) may become fully depleted and CZTS acts as back surface field layer. We analyze the prospect and performance of the proposed architecture through rigorous optoelectronic simulations. We also study the role of the Schottky barrier at the back-contact interface of a conventional CZTS cell. In this regard, we propose to use an Ohmic contact to increase the open circuit voltage by replacing the molybdenum (Mo) with indium tin oxide (ITO). We further optimize the ACZTS thickness and calculated a maximum obtainable efficiency of 17.59% at 550 nm ACZTS with 940 mV open circuit voltage, 24.65 mA cm^−2^ short circuit current and 75.94% fill factor including the effects of Shockley-Read-Hall, radiative and surface recombination mechanisms. The efficiency of the optimized cell is ∼6.6% higher than that of the existing best single junction kesterite cell. We also vary the minority carrier life time (*τ*_c_) and surface recombination velocity of back contact (SRV_back_) and report an ideal efficiency of 22.14% with *τ*_c_ = 1 μs and SRV_back_ = 1000 cm s^−1^. Finally, we replace the toxic CdS buffer layer with eco-friendly ZnS and observe a relative improvement of 12.91% in the efficiency. The concept proposed and analyses performed in this work advance the efficiency of single junction kesterite solar cells.

## Abstract 2

A CdS/ACZTS/CZTS single junction kesterite solar cell with 17.59% efficiency.

## Graphical abstract

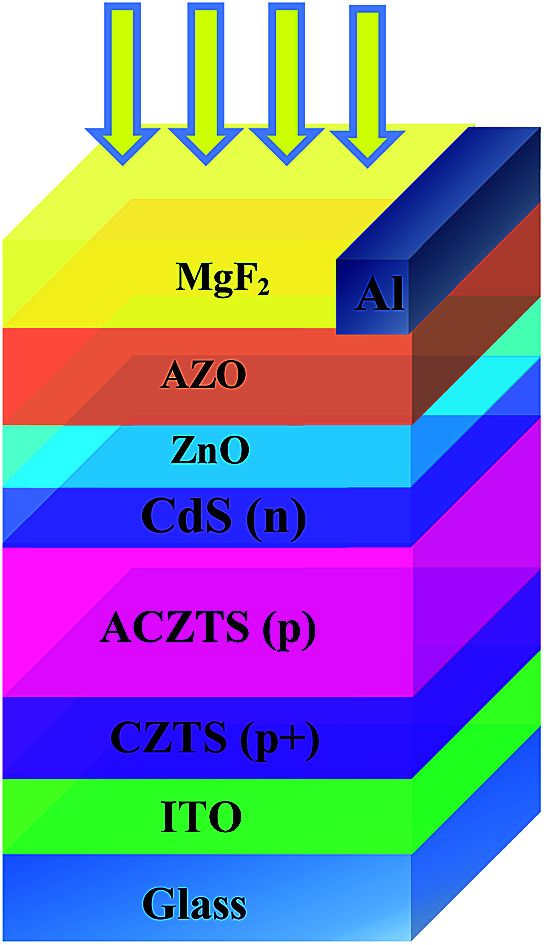

## I. Introduction

Energy harvesting through thin film solar cells is one of the emerging fields in photovoltaics (PV) due to their high power conversion efficiency (PCE), direct and tunable bandgap, reduced material usage as well as lower deposition cost on large areas.^1,2^ Although CuIn_1−*x*_Ga_*x*_Se_2_ (CIGS) based solar cells hold the record efficiency of 22.6% (ref. 3) among thin film solar cell technologies, the use of costly rare-earth materials such as Indium (In) and Gallium (Ga) may limit their production in future. In this respect, CZTS is a promising absorber layer for chalcogenide solar cells that can be used as a substitute of CIGS absorber. In CZTS, In and Ga are replaced by cheap and earth-abundant Zinc (Zn) and Tin (Sn), respectively which makes the cell cost efficient.^4,5^ Moreover, they exhibit high absorption coefficients (>10^4^ cm^−1^) and tunable bandgap properties enabling effective absorption of incident radiation within a few microns thickness of the absorber.^6–8^ However, the PCE and open circuit voltage (*V*_oc_) of CZTS solar cells are known to be limited by bulk defects, secondary phase formation, grain boundaries, CZTS/CdS and CZTS/Mo interfaces.^4,6^ The highest recorded efficiency in CZTS solar cell has been reported to be 11% with an open circuit voltage of 730.6 mV.^9^ Therefore, significant research is still needed in this field to compete with CIGS based solar cells.

Recently, Vermang *et al.* reported 32% relative improvement (5.5% from 4.1%) in the PCE of CZTS solar cell using Al_2_O_3_ rear surface passivation layer with nano-size point openings to reduce rear surface recombination and the impact of secondary phase segregation.^10^ Nonetheless, shorter diffusion length of minority carriers in CZTS and higher series resistance limit the PCE of their reported cell. In another attempt, interfacial microstructure and chemistry of CdS/CZTS heterojunction were studied by Liu *et al.*^11^ They increased the minority carrier life time by eliminating interfacial defects with chemical bath deposition (CBD) process. However, disposal and waste recycling in CBD process are disadvantageous for the fabrication industry.^12^ Apart from these, different types of buffer layer such as sputtered CdS,^13^ In_2_S_3_/CdS hybrid layer,^14^ Zn_1−*x*_Cd_*x*_S^15^ have also been proposed. Yan *et al.* reported enhancement of *V*_oc_ using double CZTS layer stacks.^16^ Nevertheless, increase of shunt resistance and reduction of optical absorption for long wavelength photons reduce the short circuit current density (*J*_sc_) and consequently the PCE of their reported cell. Previously, we proposed a dual junction architecture based on CZTS (top cell) and CZTSe (bottom cell), and reported a tandem cell with 19.87% efficiency theoretically.^17^ In a more recent effort, we proposed a triple junction environmental friendly CBTSSe/CZTS/ACZTSe tandem solar cell and optimized the device structure to achieve a maximum efficiency of 36.04%.^18^ However, the tandem cell may suffer from practical limitations such as complexities in fabricating the tunneling junction, tunneling junction losses, high material usage and thus relatively higher fabrication cost. Thereby, despite noteworthy works have been done in recent years to increase the PCE of CZTS solar cells, further improvements are required to make them commercially viable alternatives.

With the aim of improving the performance of CZTS solar cells by manipulating their carrier collection mechanism, we have designed an n/p/p+ kesterite solar cell and investigated its prospect and performance where CdS, silver (Ag) mixed CZTS [(Ag_*x*_Cu_1−*x*_)_2_ZnSnS_4_] (ACZTS) and CZTS layers have been used for n-doped, p-doped and p+-doped regions, respectively. In our design, the collection efficiency of minority carriers is enhanced by extending the depletion region throughout the entire ACZTS layer. We have performed extensive analysis of the proposed configuration to obtain an optimized structure for maximum PCE. We have also analyzed the effects of back-contact type (Schottky or Ohmic), CdS buffer layer as well as the impact of minority carrier life time and surface recombination velocity (SRV) of the back contact.

## II. Device structure and simulation methodology

**Fig. 1:**
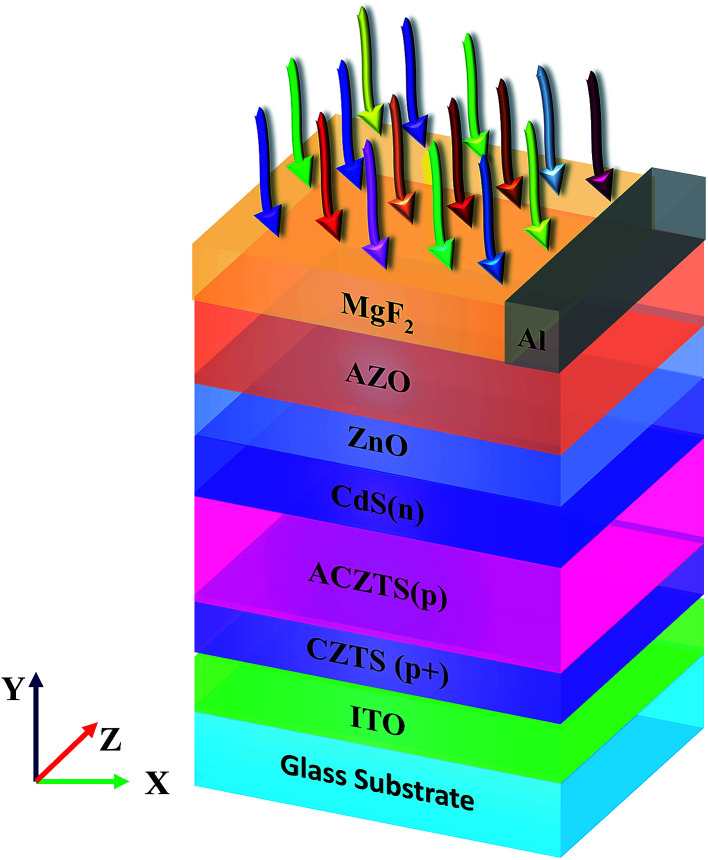
Architecture of the proposed CdS/ACZTS/CZTS (n/p/p+) configuration solar cell.

Fig. 1 shows our proposed n/p/p+ configuration of CZTS solar cell. The design is based on an experimentally demonstrated n–p structure (MgF_2_/AZO/ZnO/CdS(n)/CZTS(p)/molybdenum (Mo)/glass substrate) reported by Shin *et al.*^8^ where an additional layer of ACZTS is inserted between CZTS/CdS. Also, the back contact, Mo is replaced by indium tin oxide (ITO). Therefore, the proposed cell consists of MgF_2_/AZO/ZnO/CdS(n)/ACZTS(p)/CZTS(p+)/ITO/glass layers (Fig. 1) (In later part of our analysis, we have replaced CdS(n) buffer layer with ZnS(n) layer). Here, ACZTS and CdS work as the primary absorbing layer (p type) and buffer layer (n type), respectively. Additionally, CZTS (p+ layer) and n-type transparent ZnO window layer are applied as back and top surface field layers, respectively. Aluminum doped ZnO (AZO) is used to reduce the series resistance of the cell.^17^

The thickness of AZO, ZnO, CdS(n), CZTS(p+) are 400, 50, 100, 200 nm, respectively. We varied the thickness of ACZTS(p) in this study to maximize the efficiency of our structure. ITO on glass substrate is used as the back contact whereas Al contact with 10% coverage is used (unless otherwise stated) as the front electrode^19^ (we also varied front electrode coverage to see its effect on the maximum attainable efficiency). A 100 nm thick magnesium fluoride (MgF_2_) is placed on top of AZO as an antireflection coating layer. As far as the fabrication viability of the proposed structure is concerned, it was shown that substituting Cu with Ag causes variation in carrier density and ∼1 × 10^16^ cm^−3^ (p+ CZTS)^20^ and ∼1 × 10^14^ cm^−3^ (p ACZTS, at 7 mol% of Ag to Cu)^21^ doping in CZTS material system were reported previously (Ag in CZTS is used to vary the carrier concentration in CZTS material system and improve the absorber bulk quality by reducing the problem of band tailing^21–24^). Similarly, CZTS/ITO interface is also experimentally realizable.^25,26^ Apart from these modifications, Shin *et al.* demonstrated the fabrication of other layers in their reported cell.^8^ Therefore, the proposed structure is compatible with existing fabrication technologies. It should also be noted that extension of depletion region in conventional solar cells is usually achieved by inserting an intrinsic layer between p and n regions. However, intrinsic CZTS has not been reported yet in literature. Since the depletion width is known to be inversely proportional to doping concentration^27^ and p-type CZTS with a range of carrier concentrations have been reported,^8,20,21^ we used a relatively lower concentration p-type layer in our design to extend the depletion region.

To find the characteristics features of the proposed cell, we solved the Poisson's equation, drift–diffusion equations and continuity equations self consistently for electrons and holes [eqn (1)–(5)].

1 −∇(*ε*_dc_∇*V*) = *qρ*,

2
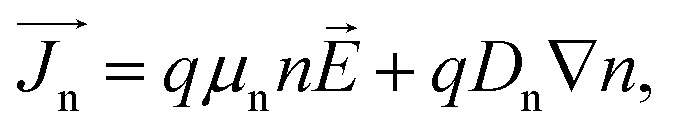
3
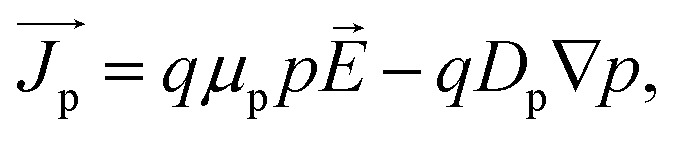
4
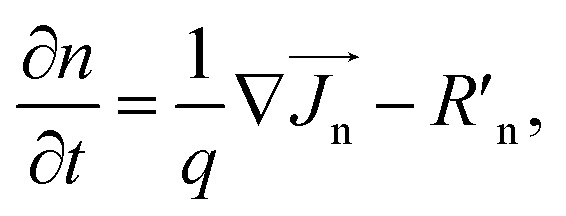
5
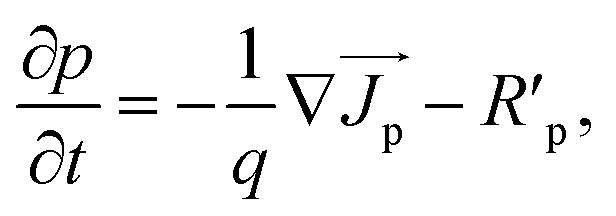
where *ε*_dc_ is the dc dielectric permittivity, *V* is the electrostatic potential (electric field, *E→* = −∇*V*), *ρ* is the net charge density (*ρ* = p − n + *C*, which includes the contribution *C* from the ionized impurity density), 
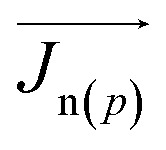
 is the electron (hole) current density, *q* is the positive electron charge, *μ*_n(p)_ is the mobility of electron (hole), *D*_n(p)_ is the diffusivity of electron (hole) 
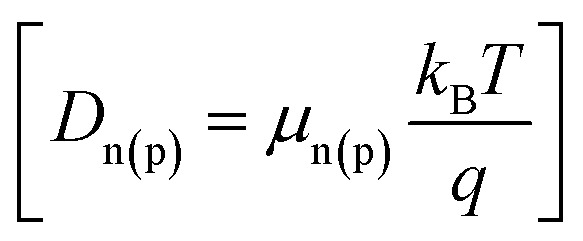
, n and p are electron and hole densities, respectively, *k*_B_ is the Boltzmann constant and *T* is the temperature, *R*′_n(p)_ is the net recombination rate (the difference between the recombination rate and generation rate) (the subscripts n and p indicate quantities that are specific to the carrier type). We considered Shockley-Read-Hall (SRH) recombination mechanism for bulk defects, surface recombination mechanism to model the surface properties and radiative recombination mechanism to account for the direct bandgap nature of the CZTS, ACZTS and CdS. The generation rate was calculated from the optical simulation. We first solved Maxwell's curl equation for optical electric field 
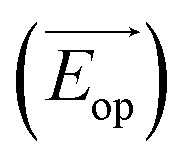
 distribution. Each material was modeled by their respective refractive index (*n*) and extinction coefficient (*κ*) as a function of wavelength. The absorbed power (*P*_abs_) inside different layers can be calculated from the optical electric field [eqn (6)].6
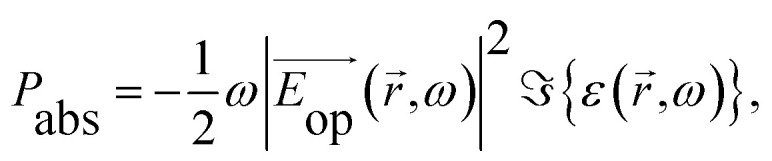
where *ω* is the angular frequency and *ε*(*r⃑*,*ω*) is the dielectric constant. Thus, the generation rate, *G*(*r⃑*) was calculated from the eqn (7) and (8).7
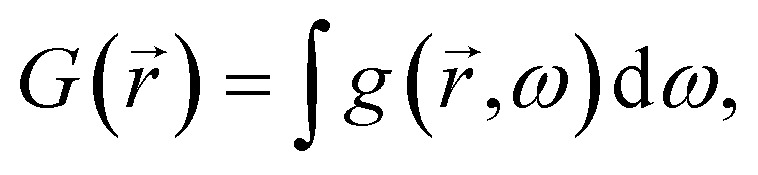
8
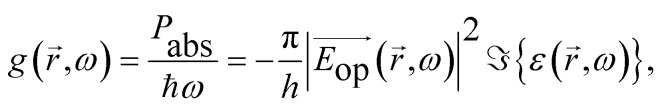
where *h* is the Planck's constant.

For optical simulations, periodic boundary condition was used in horizontal (*X*) direction and perfectly matched layer (PML) boundary condition was applied on top and bottom faces (in *Y*-direction). AM 1.5 G standard solar spectrum has been used as the input radiation source. Lumerical Device and FDTD solution softwares have been used for implementing the model equations.

## III. Results and discussion

### A. Model verification

**Fig. 2:**
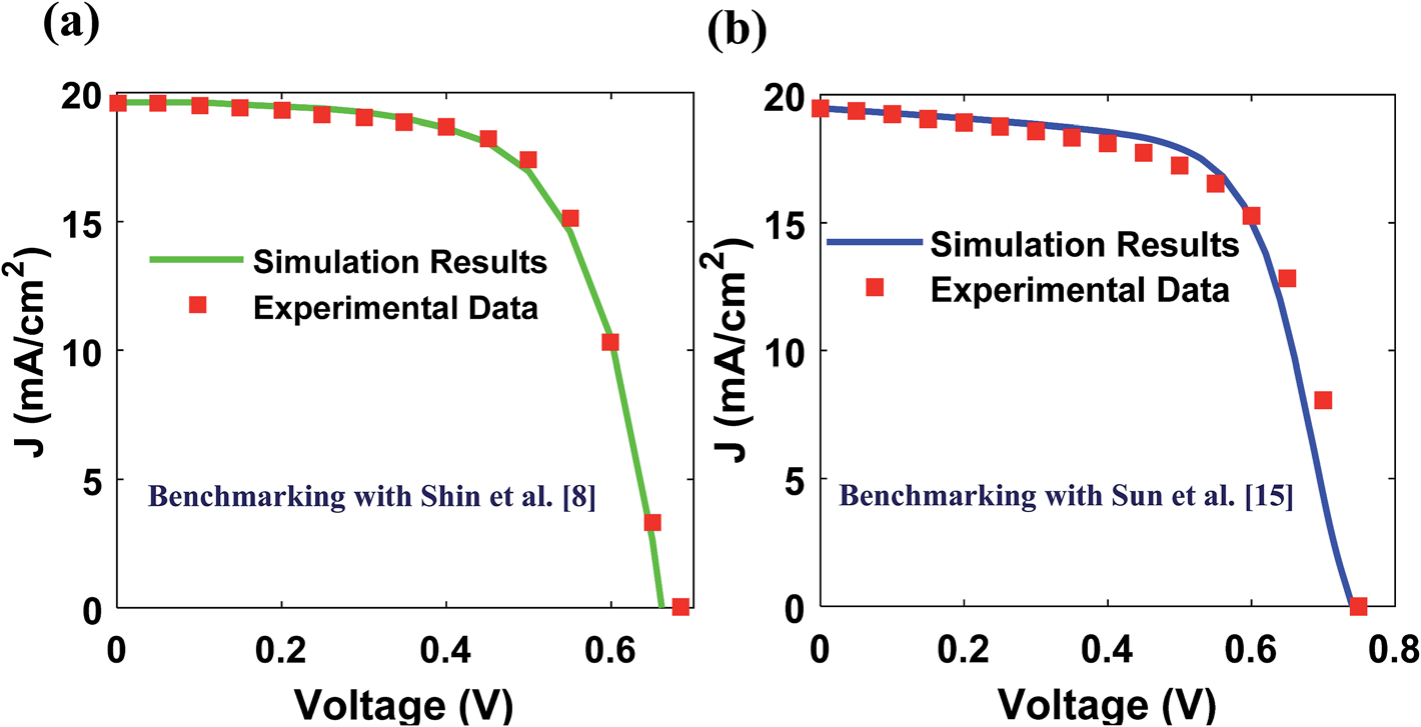
Comparison of *J*–*V* characteristics obtained from our simulation with the experimental results of (a) Shin *et al.*^8^ and (b) Sun *et al.*^15^

We started with modeling the existing experimental structure reported by Shin *et al.*^8^ and Sun *et al.*^15^ to benchmark our used parameters and simulation environment. Fig. 2 and Table 1 show the *J*–*V* characteristic and comparison between our simulation and experimental results,^8,15^ respectively. The optical and basic electrical parameters as well as recombination parameters were taken from literature (optical;^20,21,28–30^ electrical^8,17,19–24,31–38^ (listed in Table 2)). In this respect, we have used minority carrier life time of CZTS 0.47 ns and 0.72 ns for matching with the experimental results of Shin *et al.*^8^ and Sun *et al.*^15^ respectively. It can be noted from Table 1 that the performance metrics obtained from simulation have good agreement with the experimental data. As we matched the experimental results of latest highest performance device reported by Sun *et al.*^15^ with 0.72 ns minority carrier life time of CZTS, we have used that value in our simulations.

**Table 1:** Comparison of characteristics features between the experimental data^8^ and our simulation results

	*V*_oc_ (mV)	*J*_sc_ (mA cm^−2^)	FF (%)	Efficiency (%)
**Comparison with Shin *et al.*** ^**8**^				
Experimental^8^	661	19.5	65.8	8.4
Simulation	678	19.63	65.3	8.68
**Comparison with Sun *et al.*** ^**15**^				
Experimental^15^	747	19.5	63.2	9.2
Simulation	740	19.45	64.5	9.28

**Table 2:** Basic parameters used in simulations

Features	CZTS (p+)^8,17,19,20,31^	ACZTS (p)^21–24^	CdS (n)^17,19,32,33^	ZnS (n)^17,32,35^	ZnO (n+)^17,32,36–38^	AZO (n++)^17,32,36–38^
DC permittivity	7	7	10	9	9	9
Bandgap (eV)	1.45	1.5	2.42	3.58	3.37	3.37
Electron affinity (eV)	4.1	4.14	3.75	3.8	4	4
Electron effective mass (*m*_e_/*m*_o_)	0.18	0.18	0.25	0.22	0.275	0.275
Hole effective mass (*m*_p_/*m*_o_)	2	2	5	1.76	0.59	0.59
Electron mobility (cm^2^ V^−1^ s^−1^)	30	1	160	230	150	50
Hole mobility (cm^2^ V^−1^ s^−1^)	25	0.9	15	40	50	5
Acceptor concentration (cm^−3^)	1 × 10^16^	1 × 10^14^	0	0	0	0
Donor concentration (cm^−3^)	0	0	5 × 10^16^	5 × 10^16^	1.5 × 10^17^	8 × 10^18^
SRH life time (s)	7.2 × 10^−10^	7.5 × 10^−10^	7.5 × 10^−10^	5.5 × 10^−10^	—	—
Radiative recombination (ehp capture rate cm^3^ s^−1^)	1.04 × 10^−10^	1.1 × 10^−10^	1.02 × 10^−10^	1.5 × 10^−10^	—	—
Surface recombination velocity (cm s^−1^)	1 × 10^7^ (back surface/CZTS)	—	—	—	—	1 × 10^7^ (AZO/Al and AZO/MgF_2_)

**Fig. 3:**
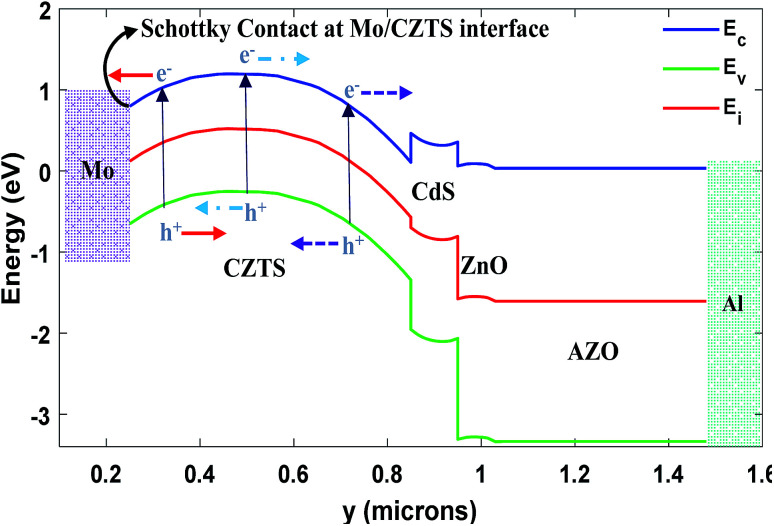
Band diagram of the reported CZTS cell by Shin *et al.*^8^ where dotted arrows indicate drift and diffusion of carriers toward correct electrodes, red solid arrow indicates motion of carriers toward wrong electrodes. Wrong motion of carriers would be subjected to recombination in absence of proper surface field layers.

The PCE of conventional CZTS solar cell reported by Shin *et al.* is only 8.4%. Thus, we investigated the limiting factors behind the performance of their CZTS solar cell having layers of MgF_2_/AZO/ZnO/CdS(n)/CZTS(p)/Mo/glass substrate (top to bottom).^8^ We have done this by analyzing the band diagram of their cell (Fig. 3). The energy band diagram has been drawn from our simulation results. A Schottky contact can be noticed at the Mo/CZTS interface. The Schottky contact arises from the fact that the work function of Mo (4.6–4.9 eV)^39^ is less than that of CZTS (∼5.15 eV).^31^ This reduces the built-in-potential of the cell and consequently increases the dark current. Thus, *V*_oc_ also decreases since it is inversely proportional to the logarithm of dark saturation current.^40^ Moreover, due to the band bending around Mo/CZTS interface (Fig. 3), optically generated minority carriers in CZTS are attracted to Mo interface and subjected to surface recombination which reduces the *J*_sc_. Therefore, apart from formation of MoS_2_ and reaction with CZTS absorber (responsible for instability and reduction in effective absorber layer thickness^4,41^), Mo contact also lowers both *V*_oc_ and *J*_sc_ of CZTS solar cell, and hence the overall PCE of the cell. Furthermore, we noticed that there was no back-surface-field (BSF) layer^8^ and a conduction band-offset at CdS/CZTS interface acts as an obstacle for optically generated minority carriers (electrons) in CZTS to get transported into CdS(n) region.

In this regard, as Shin *et al.* reported spike-type CdS/CZTS interface,^8^ we considered spike-like conduction band offset (CBO) at CdS/CZTS in our band diagram. Moreover, the electron affinity of CdS(∼3.75 eV)^17,19^ is lower than that of CZTS (∼4.1 eV)^19^ which makes the CBO at CdS/CZTS (Δ*E*_c_) positive, ensuring the spike-like CBO at CdS/CZTS interface. Although, the band alignment of CdS/CZTS in lower performance devices has been reported to be cliff-type which limits the *V*_oc_ of solar cell by assisting interfacial recombination,^5,42,43^ Crovetto *et al.* recently reported that best performance devices (*η* > 8%) have spike-like CdS/CZTS interface.^44^ Apart from that, Rondiya *et al.* studied complete CV analysis for band edge position with band offset calculations for spike-like CdS/CZTS interface.^45^ Additionally, Kaur *et al.* presented CBO variation for different S/(S + Se) ratio^46^ and it was found that spike-like offset is present for all the cases of S/(S + Se) ratio^46,47^ which justifies our spike-like CdS/CZTS interface.

Based on the above observations, we have modified the structure of Shin *et al.* as follows. We used ITO in place of Mo, as back contact, whose work function can be tuned and matched to the work function of CZTS by varying its doping. Thus, the undesired Schottky contact with CZTS layer can be eliminated. In fact, ITO with work function of 5.25 eV has been reported in literature which is suitable to form ohmic contact with CZTS.^48–50^ Next, we chose CdS/ACZTS/CZTS (n/p/p+) layers in which the built-in-potential originates from CdS/ACZTS (n/p) layers and CZTS (p+) is used as BSF layer. Due to the lower carrier concentration in ACZTS (∼10^14^ cm^−3^ at 7 mol% of Ag to Cu)^21–24^ compared to CdS (∼10^17^ cm^−3^),^19^ the depletion layer in ACZTS (p) region can be much wider than the reported effective thickness (∼500 nm) of CZTS solar cell.^8^ It was calculated to be more than one micron for 1 × 10^14^ cm^−3^ and 5 × 10^16^ cm^−3^ doping in ACZTS(p-region) and CdS (n-region), respectively [from eqn (9)^27^].9
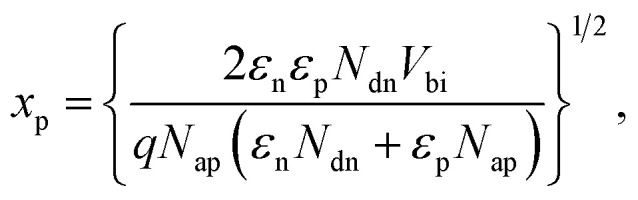
where *ε*_n_ (*ε*_p_) is the permittivity, *N*_dn_ (*N*_ap_) is the doping concentration of n (p) region and built-in potential *V*_bi_ can be calculated as10
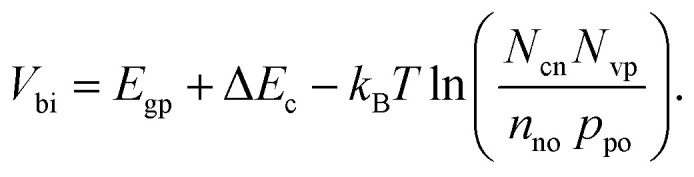


Here, *E*_gp_ is the bandgap of p-region, Δ*E*_c_ is the difference between electron affinity of n and p regions, *N*_cn_ is the density of state (DOS) of electron in n region and *N*_vp_ is the DOS of hole in p region, *n*_no_ and *p*_po_ are the equilibrium concentration of electrons and holes in n and p regions, respectively.

The typical carrier concentration in CZTS (∼10^16^–10^17^ cm^−3^) is several orders higher than that of ACZTS (∼10^14^ cm^−3^).^19–21^ Thus, the CZTS layer acts as p+ BSF layer and reduces rear surface recombination. A much lesser minority carrier life time of ACZTS and CZTS (0.75 ns and 0.67 ns, respectively) was used in our simulation than the reported values in literature (∼7 ns)^8,21^ in order to predict the minimum PCE of our designed cell. We also varied the lifetime in the later part of our analysis to observe its effect and predict the prospect of our proposed cell for absorber layers having higher diffusion lengths.

**Fig. 4:**
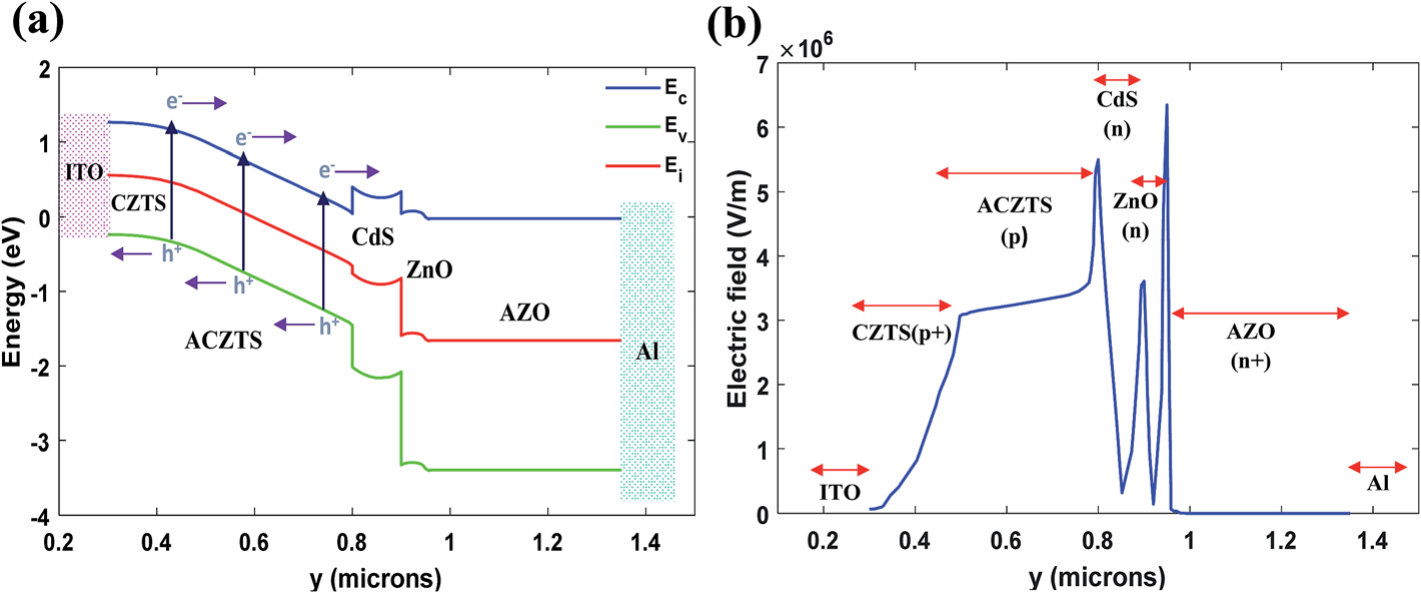
(a) Energy band diagram and (b) electric field distribution of our proposed structure (at 300 nm and 200 nm thickness of ACZTS and CZTS, respectively). Arrows in (a) represent drift motion of carriers.

As mentioned earlier, effective thickness of CZTS reported by Shin *et al.*^8^ was around 500 nm, we started our analysis with reasonable thicknesses of the primary absorber and buffer layer (300 nm thick ACZTS (p-region) and 200 nm thick CZTS (p+-region)). We found an efficiency of 16.08% with *V*_oc_ = 939 mV, *J*_sc_ = 22.44 mA cm^−2^ and FF = 76.12%. Fig. 4(a) and (b) show the band diagram and electric field distribution of the proposed n/p/p+ structure, respectively. It can be seen from the electric field distribution [Fig. 4(b)] that the depletion region is extended throughout the entire ACZTS region, as predicted from our calculation [eqn (9) and (10)]. The extension of depletion region throughout the n/p/p+ regions and the reduction of surface recombination due to the BSF layer increase the collection probability of generated carriers leading to high current density in our modified configuration. Extended field distribution helps the generated minority carriers to be efficiently collected by the built-in electric field through drift mechanism. Moreover, ITO as back contact eliminates the drawback of band bending which significantly improves the open circuit voltage of the cell. Therefore, the PCE is higher in our designed solar cell.

### B. Optimization of ACZTS thickness

**Fig. 5:**
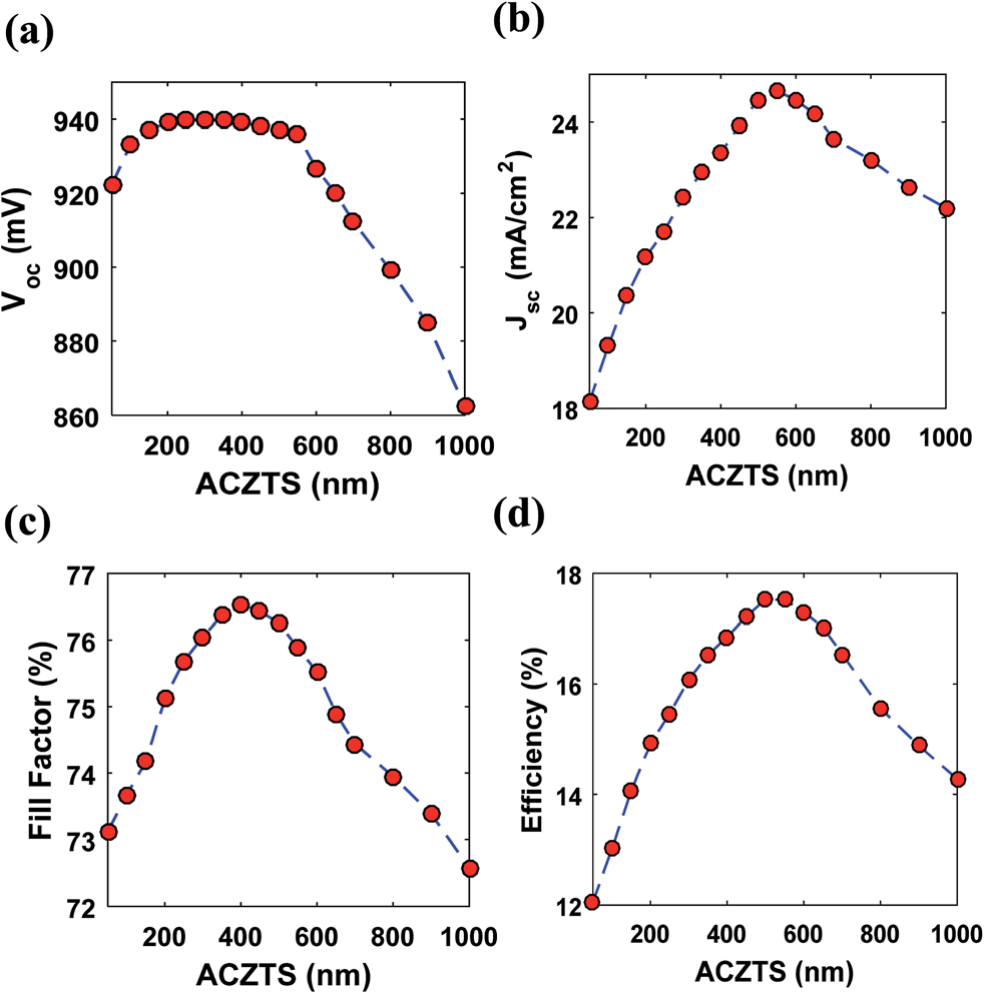
Variation of (a) open circuit (*V*_oc_), (b) short circuit current density (*J*_sc_), (c) fill factor (FF) and (d) efficiency (*η*) with the thickness of ACZTS.

**Fig. 6:**
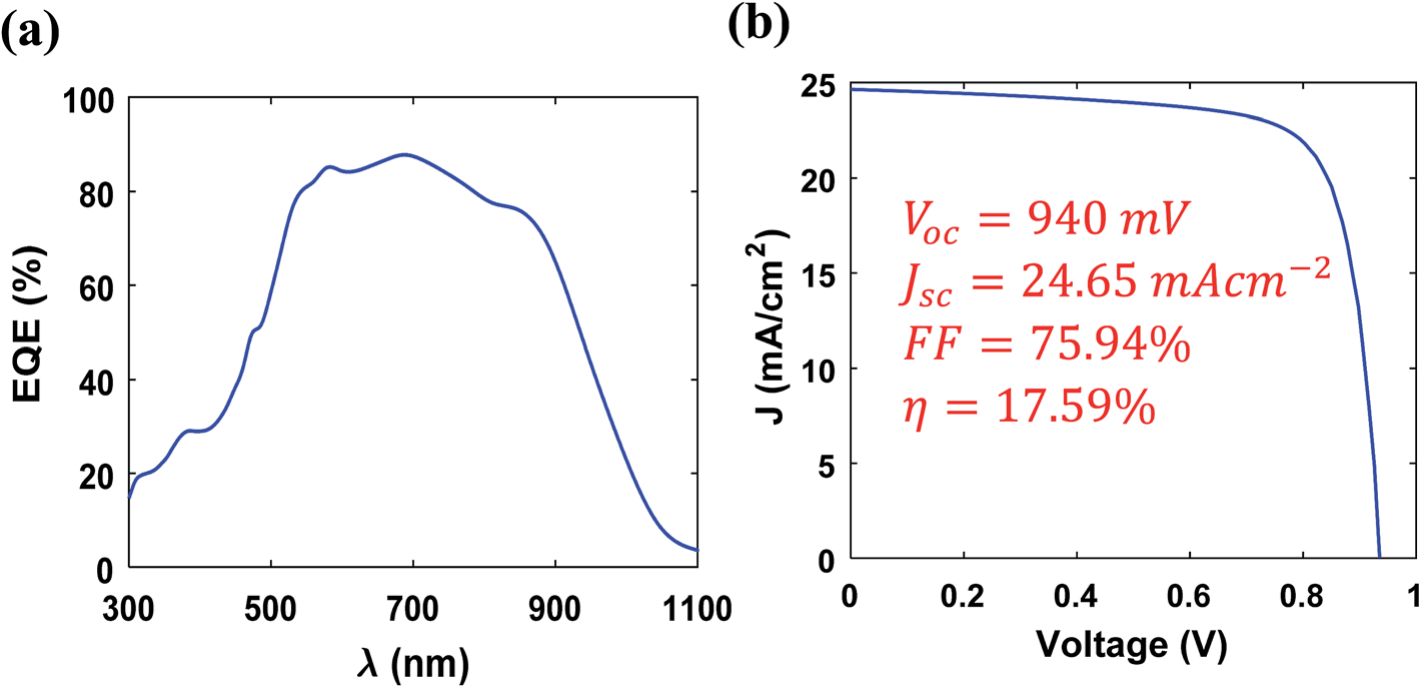
(a) EQE and (b) *J*–*V* characteristics of the optimized cell at 550 nm ACZTS.

In this study, we varied the thickness of ACZTS (p-region) in order to find an optimum thickness for which the efficiency maximizes. Fig. 5 shows the variation of *V*_oc_, *J*_sc_, FF and PCE (*η*) with the thickness of ACZTS. First, we analyze the dependence of *V*_oc_ on the thickness of ACZTS [Fig. 5(a)]. It is known that *V*_oc_ of a solar cell is logarithmically proportional to the optical generation of carriers.^51^ Since generation increases with the thickness of ACZTS, *V*_oc_ is also expected to increase with the thickness. However, *V*_oc_ increases monotonically between 50 and 300 nm and remains almost constant in the region between 300–550 nm due to its logarithmic dependence. For further increase in thickness, the recombination increases significantly which reduces the *V*_oc_ in the higher thickness region. The highest *V*_oc_ obtained at 300 nm thickness of ACZTS is 940 mV and lowest for the simulated range is found at 1000 nm (862 mV). It is also important to investigate the dependence of *J*_sc_ and FF on the thickness of ACZTS to find the overall impact on the maximum obtainable PCE [Fig. 5(b) and (c)]. As can be seen from Fig. 5(b), *J*_sc_ increases sharply with the thickness of ACZTS in the region between 100 and 550 nm due to proportionally increased optical generation.^40^ At 550 nm thickness of ACZTS, maximum value of *J*_sc_ is obtained (24.65 mA cm^−2^). Beyond that, recombination mechanisms play dominant role and the current density decreases similar to *V*_oc_. Lastly, FF of the cell increases up to 400 nm of ACZTS and then decreases due to the combined effect of *V*_oc_ and *J*_sc_ [Fig. 5(c)]. The overall variation of the efficiency with the thickness of ACZTS is shown in Fig. 5(d). Since the variation of *V*_oc_ and FF is relatively smaller than the variation in *J*_sc_ up to 550 nm, the overall efficiency resembles the trend of *J*_sc_. The downward trend of the efficiency seems to be a combined effect of decreasing *J*_sc_, *V*_oc_ and FF. We found that a maximum efficiency of 17.59% can be achieved at the thickness of 550 nm of ACZTS (with 200 nm thickness of CZTS). Then, we also varied the thickness of CZTS (BSF layer) to see its effect on PCE. Table 3 represents the variation of *V*_oc_, *J*_sc_, FF and *η* with CZTS thickness. It can be seen that BSF layer reduces the surface recombination and increases the overall PCE of the cell, as expected, by improving the *V*_oc_, *J*_sc_ and FF. Increasing thickness of CZTS improves the built-in potential and consequently the *V*_oc_. Moreover, it slightly improves *J*_sc_ due to higher absorption. However, recombination mechanism dominates at higher thicknesses of CZTS which reduces the *V*_oc_ and *J*_sc_ after 200 nm. Therefore, PCE is optimized at 200 nm thickness of CZTS and our analysis with 200 nm BSF layer is reasonable. Fig. 6(a) illustrates the external quantum efficiency (EQE) for 550 nm ACZTS and 200 nm CZTS. We calculated a collection efficiency of over 80% in the wavelength range between 550 and 850 nm. Fig. 6(b) represents the corresponding *J*–*V* characteristics at the optimized thickness.

**Table 3:** Variation of performance metrics (*V*_oc_, *J*_sc_, FF and *η*) of our modified structure with the thickness of CZTS (BSF layer)

Thickness of CZTS (BSF layer) (nm)	*V*_oc_ (mV)	*J*_sc_ (mA cm^−2^)	FF (%)	Efficiency, *η* (%)
No BSF	801	21.20	75.75	12.85
50	913	23.58	76.89	16.55
100	924	23.82	76.34	16.80
150	930	24.20	76.08	17.12
200	940	24.65	75.94	17.59
250	925	24.25	75.65	16.97
300	915	24.16	75.46	16.68

It is noteworthy to point out that since ACZTS is a new material in photovoltaic arena, the effective masses of electron and hole, and electron mobility for ACZTS haven't yet been reported. Thus, we have varied these parameters within a wide range to check the validity of our assumed values. It was found that the variation in the PCE of the optimized cell is <1% for the variation of hole effective mass within 0.5–2.5 (*m*_h_/*m*_o_), electron effective mass within 0.18–0.8 (*m*_e_/*m*_o_) and electron mobility within 1–100 cm^2^ V^−1^ s^−1^. The detail analysis is given in the ESI.† Generally, it is expected that the mobility of electrons in ACZTS should be higher than that of CZTS since the doping is lower in ACZTS. Thus, one can expect an increase of around ∼0.8% in the PCE of the proposed cell for higher mobility of electrons, as shown in the supplementary analysis. Nonetheless, we assumed a low value of electron mobility in ACZTS to predict the minimum performance of our cell.

It can also be noted that although we have considered 10% Al coverage in our simulations,^19^ front electrode coverage has been reported to vary in-between 5% to 10% of the device area.^8,19,52^ Thus, we have also varied the percentage of front electrode coverage area in our optimized structure and Table 4 summarizes its effect on the characteristics features. As expected, reduced front electrode coverage decreases the shading optical losses and consequently raises the *J*_sc_ of the cell. Further, due to the logarithmical dependence with optically generated photons, *V*_oc_ of the cell also rises with lower front electrode coverage and as a result, we expect that the efficiency will further increase with reduced front electrode coverage. At 5% Al coverage, a maximum efficiency of 19.21% is obtained with *V*_oc_ = 970 mV, *J*_sc_ = 25.87 mA cm^−2^ and FF = 76.59%.

**Table 4:** Variation of *V*_oc_, *J*_sc_, FF and *η* of our modified structure with front electrode coverage area at 550 nm of ACZTS

Area coverage of front electrode (Al) (%)	*V*_oc_ (mV)	*J*_sc_ (mA cm^−2^)	FF (%)	Efficiency, *η* (%)
10	940	24.65	75.94	17.59
9	948	24.92	76.08	17.97
8	955	25.15	76.15	18.29
7	962	25.39	76.29	18.64
6	966	25.64	76.45	18.94
5	970	25.87	76.59	19.21

Another issue worth discussing is that high quality CZTS absorber usually requires high temperature sulfurization process which can reduce the crystalline quality of ITO leading to degradation of conducting performance.^25,53^ For this reason, we have done simulation considering high sheet resistance of ITO (∼43 Ωcm^−2^ at 560 °C which is five times higher than original sheet resistance of ITO) reported by Tao *et al.*^53^ and found that FF can be degraded around ∼8% (75.94% to 70.18%) due to high sheet resistance. For this case, the maximum efficiency at 550 nm of ACZTS is found to be 15.65% with *V*_oc_ = 908 mV, *J*_sc_ = 24.52 mA cm^−2^ and FF = 70.18% which is ∼11% lower than our previously obtained maximum efficiency. However, it can be noted that Ge *et al.* used sulphur vapor to grow CZTS at higher temperature without significantly increasing the sheet resistance of ITO and the contribution of ITO back contact resistance to series resistance of the overall cell was found negligible.^26^ Moreover, they also found that ITO remains highly conductive after high temperature annealing process which validates the viability of ITO as the back contact of a CZTS architecture. However, increment of annealing temperature and duration may cause Sn atoms to be displaced by In which results the formation of indium incorporated CZTS (CZTIS). This secondary phase may result higher density lattice defects,^25^ lower symmetry of crystalline structure^25^ and severe band tailing^26^ which can degrade the performance of CZTS solar cell. On the other hand, hole density in CZTIS (∼10^19^ cm^−3^) is three order higher than that of pure CZTS (∼10^16^ cm^−3^) which can boost the built-in potential and thus the *V*_oc_ of the device.^25^

### C. Effect of SRH life time and back surface recombination velocity

Since electric field inside the p-region (ACZTS) decays with thickness, minority carriers (electrons) at the bottom end of the p-region (ACZTS) get an opportunity to recombine with majority carriers (holes) which decreases the collection probability of minority carriers. If one can reduce the defect density by improving crystal growth techniques, the minority carrier life time will increase and accordingly, collection probability of minority carriers will rise. Moreover, at high SRV of the back contact (SRV_back_), minority carriers have higher probability of being subjected to back surface recombination. As the lattice parameters of ITO and CZTS match approximately,^26^ the interfacial states between ITO/CZTS interface are expected to decrease. As a result, SRV would be low and minority carriers near back contact would have a chance to diffuse back in the p-region (ACZTS) where they would be drifted by the built-in electric field towards the correct electrode. To show the effect of these parameters, we varied the SRH recombination life time and SRV of rare (back) contact at the optimized thickness.

**Fig. 7:**
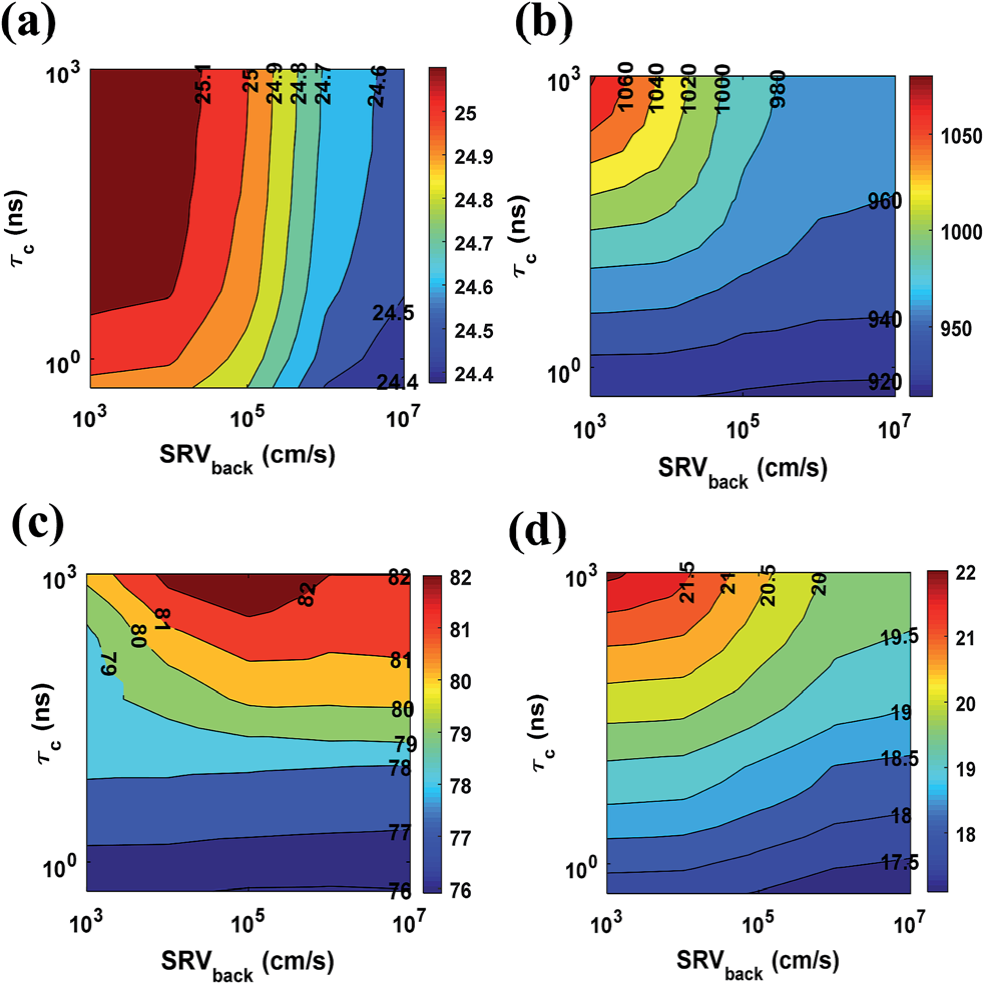
Variation of (a) short circuit current (*J*_sc_) (b) open circuit voltage (*V*_oc_) (c) fill factor (FF) and (d) efficiency (*η*) with SRH recombination life time (*τ*_c_) and SRV of the back contact (SRV_back_).

Fig. 7 represents the variation of *V*_oc_, *J*_sc_, FF and *η* with minority carrier life time (*τ*_c_) and SRV of the back contact (SRV_back_). *J*_sc_ is found to be maximum at high *τ*_c_ and low SRV_back_ region [Fig. 7(a)], as expected. Additionally, reduction of recombination decreases the diode dark current and increases photon generated current.^40^ Thus, *V*_oc_ also remains higher at high *τ*_c_ and low SRV_back_ values [Fig. 7(b)]. Maximum values of *J*_sc_ and *V*_oc_ are found to be 25.18 mA cm^−2^ and 1080 mV, respectively, at *τ*_c_ = 1 μs (highest value used in simulation) and SRV_back_ = 1000 cm s^−1^ (lowest value used in simulation). We also investigated the dependence of FF with *τ*_c_ and SRV_back_ [Fig. 7(c)].

It can be seen that FF increases with carrier life time. However, it maximizes at ∼1 × 10^4^ to 1 × 10^5^ cm s^−1^ of SRV_back_. The overall effect of these parameters is reflected in the variation of PCE [Fig. 7(d)]. The maximum PCE at the highest minority carrier life time and the lowest SRV_back_ (*τ*_c_ = 1 μs and SRV_back_ = 1000 cm s^−1^) is 22.14%.

### D. ZnS as an alternative to the CdS layer

**Fig. 8:**
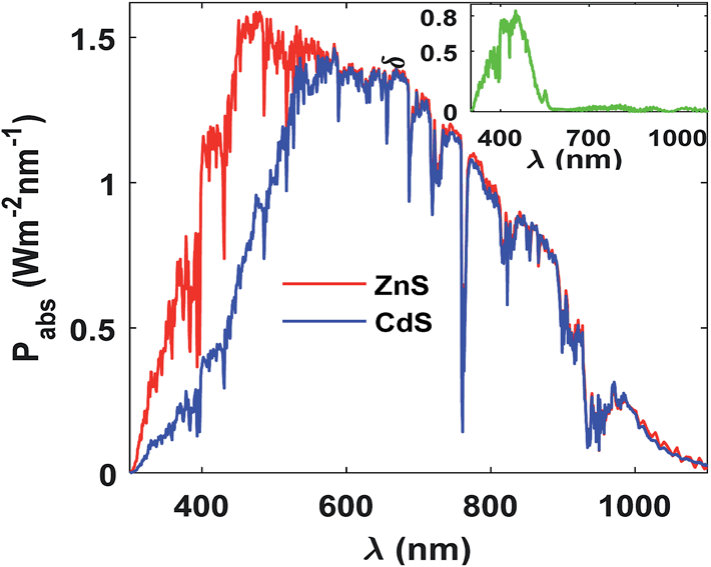
Comparison of absorbed power between ZnS and CdS buffer layers. Inset shows the difference in absorbed power for the two cases [δ = *P*_abs_(ZnS) − *P*_abs_(CdS)].

ZnS could serve as an eco-friendly and cheap buffer layer in our proposed configuration.^54^ We replaced the environment pollutant CdS buffer with ZnS and repeat the optimization steps for efficiency maximization. The optimum thickness of ACZTS was found around 700 nm in this case. ZnS has a bandgap of ∼3.58 eV which is significantly higher than that of CdS (∼2.42 eV).^32^ Therefore, high energy photons get passed through ZnS buffer layer and reach to p-region (ACZTS) where lower wavelengths of solar spectrum may get absorbed more efficiently (Fig. 8). This leads to an increment in the optimum thickness of ACZTS for the maximum efficiency. In addition to this, the conduction band offset (Δ*E*_c_) at ACZTS/buffer layer interface (Fig. 3) is reduced by 28.57% compared to the case of CdS layer (Δ*E*_c_ = 0.25 eV in case of ZnS/ACZTS while Δ*E*_c_ = 0.35 eV in CdS/ACZTS). Moreover, ZnS exhibits higher hole mobility (40 cm^2^ V^−1^ s^−1^*vs.* 15 cm^2^ V^−1^ s^−1^ in CdS) which also enhance the collection efficiency.^17,32^ All these factors contribute to the increased PCE of the proposed structure with ZnS buffer layer. Specifically, the increment in current density (7.78% relative) is due to the increased optical power absorption and reduced conduction band offset at ACZTS/ZnS interface (facilitates minority carrier collection). We also observed a relative increase of 3.72% in *V*_oc_ and a negligible change in FF. In total, 12.91% efficiency (relative) improvement was observed with ZnS buffer layer. Table 5 represents the comparison of the characteristics features between ZnS and CdS buffer layers. However, it should be noted that the CBO at ZnS/ACZTS can be as high as +0.40 eV high which could block the photo-generated minority carrier (electrons) transport resulting a decrement in carrier collection efficiency. Apart from that, strain in ZnS/ACZTS can increase the defects density which may lead to higher interfacial recombination. Additionally, secondary phase at ZnS/ACZTS can detrimentally rise the series resistance of the overall cell. All these factors may lead to a reduced fill factor and efficiency of the cell.

**Table 5:** Comparison of characteristics features between CdS and ZnS buffer layers

Buffer Layer	*V*_oc_ (mV)	*J*_sc_ (mA cm^−2^)	FF (%)	Efficiency (%)
CdS	940	24.65	75.94	17.59
ZnS	975	26.57	76.70	19.86

## IV. Conclusion

In summary, we investigated the prospect of a CdS/ACZTS/CZTS (n/p/p+) solar cell. The proposed design solves the drawback of Schottky contact at CZTS/Mo interface by introducing ITO as back contact. Doping concentrations of n and p regions ensure a fully depleted ACZTS absorber and consequently effective collection of photocarriers. We also optimized the thickness of ACZTS to find the maximum attainable PCE and 17.59% efficiency was calculated at 550 nm ACZTS and 200 nm CZTS. The calculated efficiency is ∼6.6% higher (17.59% *vs.* 11%) than the experimentally reported single junction CZTS solar cell and comparable to CZTS/CZTSe dual junction tandem cell (17.59% *vs.* 19.87%). Finally, ZnS has been proposed as an alternative to CdS buffer layer showing an increase of 12.91% (relative) in the efficiency at the optimum condition. The design and analysis presented in this paper would help kesterite solar cells to achieve an efficiency ∼20% from single junction and contribute to achieving eco-friendly, cheap inorganic solar cells.

## Conflicts of interest

There are no conflicts of interest to declare.

## Supplementary Material

RA-008-C7RA12352C-s001
