# Supplementary material for: Boosting the efficiency of single junction kesterite solar cell using Ag mixed Cu2ZnSnS4 active layer
Source: RSC Adv. 2018 Jan 29;8(9):4905–13. doi: 10.1039/c7ra12352c (PMC9078036; doi:10.1039/c7ra12352c)
Supplement: RA-008-C7RA12352C-s001 [file RA-008-C7RA12352C-s001.pdf]

Electronic Supplementary Material (ESI) for RSC Advances.
This journal is © The Royal Society of Chemistry 2018

Supplementary Information for
"Boosting the efficiency of single junction kesterite solar cell using Ag mixed Cu_2ZnSnS_4 active layer"

Uday Saha and Md. Kawsar Alam[1]
Department of Electrical and Electronic Engineering,
Bangladesh University of Engineering and Technology,
Dhaka 1205, Bangladesh.

A. Effect of effective masses of electron and hole in ACZTS

We varied the effective masses of electron and hole in ACZTS (p-type) to analyze their effect on the PCE of our designed cell (**Fig. S1**). In this regard, the range is selected from reported effective masses of kesterite compounds.[1] The collection probability of minority carriers (electrons) reduces with the increment of effective mass of electrons. Moreover, dark diode saturation current increases with the effective mass of electron which consequently decreases the V_{oc}.[2, 3] As a result, the efficiency of our designed cell decreases with effective mass of electron (**Fig. S1(a)**). Similarly, the density of states in band valance increases with the increment of effective mass of hole. This reduces the built-in potential of heterojunction solar cell which lowers the V_{oc} as well.[4] Thus, the PCE of the cell decreases with the increment of effective mass of hole (**Fig. S1(b)**). However, the variation in PCE is less than 1% for the considered range of effective masses of electrons and holes.

[1] Corresponding Author. *Email Addresses:* kawsaralam@eee.buet.ac.bd ; kawsar.alam @alumni.ubc.ca.

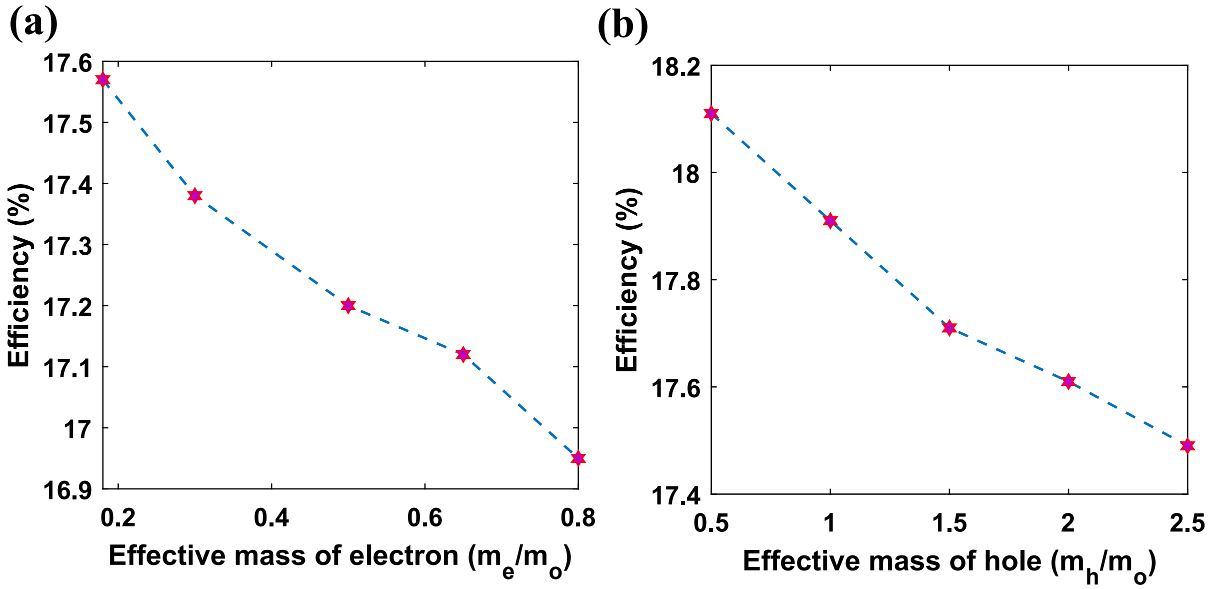

Fig S1. The effect of (a) electron's effective mass and (b) hole's effective mass of ACZTS on the efficiency of our designed cell with 550nm thick ACZTS absorber.

B. Effect of mobility of electron in ACZTS

We also observe the effect of electron mobility (μ_e) in ACZTS on the PCE of our proposed configuration. **Figure S2** represents the variation of V_{oc}, J_{sc}, *FF* and η with the electron mobility of ACZTS. As dark saturation current of solar cell increases with the rise of electron mobility, it reduces the V_{oc} (**Fig. S2(a)**). On the other hand, since minority carriers (electrons) are drifted by the built-in electric field, collection probability of minority carriers increases with the mobility of electrons. As a result, J_{sc} rises with the mobility of electron and saturates after $\mu_e = 50\ cm^2V^{-1}s^{-1}$ when most of the carriers are collected (**Fig. S2(b)**). Thus, fill factor also increases with the mobility of electron (**Fig. S2(c)**). The increment of J_{sc} and *FF* dominates the decrement of V_{oc} and as a result, an overall increase can be seen in the PCE of our proposed cell at 550 nm ACZTS **(Fig. S2(d)**). The maximum PCE is 18.42% with V_{oc}=919 mV, J_{sc} =25.05 mA/cm^2 and *FF*=80.03% at $\mu_e = 100\ cm^2V^{-1}s^{-1}$. To predict the minimum efficiency of our cell,

we have considered $\mu_e = 1\ cm^2V^{-1}s^{-1}$ in our simulations (**Table 2**), for which the efficiency is

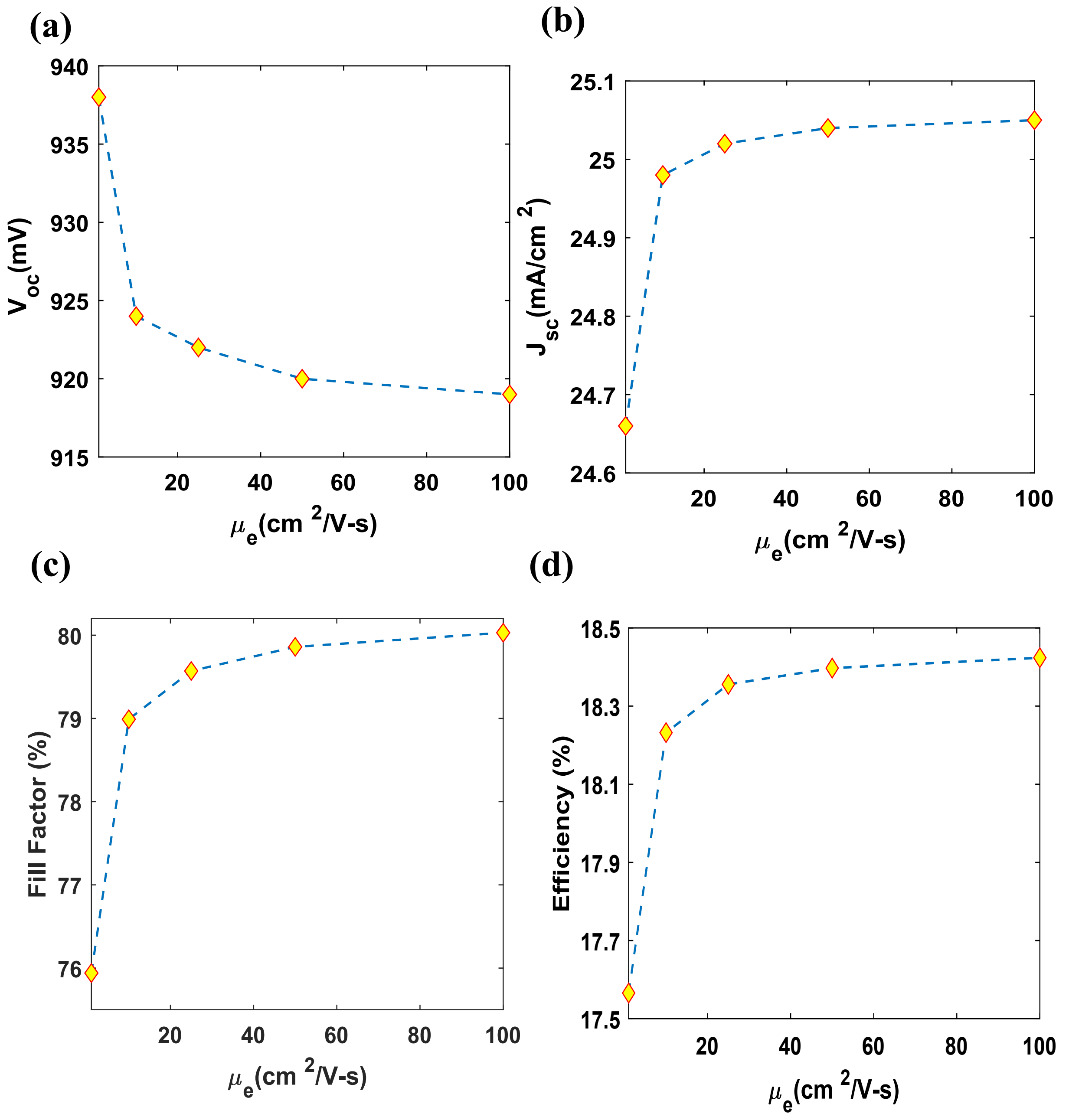

Fig S2. The variation of (a) open circuit voltage (V_{oc}) (b) short circuit current density (J_{sc}) (c) Fill Factor (FF) and (d) efficiency (η) with the electron mobility (μ_e) of ACZTS at 550 nm thick ACZTS cell.

minimum (**Fig. S2(d)**).

References

1. C. Persson, R. Chen, H. Zhao, M. Kumar and D. Huang, in *Copper Zinc Tin Sulfide-Based Thin-Film Solar Cells*, John Wiley & Sons Ltd, New Jersey, USA, 2014, 75-105.

2. S. O. Kasap, *Optoelectronics & Photonics: Principles & Practices: International Edition*, Pearson Higher Ed, London, England, 2013.

3. D. Neamen, *Semiconductor Physics And Devices*, McGraw-Hill Inc., New York, USA, 2003.

4. P. Bhattacharya and L. Y. Pang, *Semiconductor Optoelectronic Devices*, AIP, Prentice Hall, New Jersey, USA, 1994.
